# Performance of anterior nares and tongue swabs for nucleic acid, Nucleocapsid, and Spike antigen testing for detecting SARS-CoV-2 against nasopharyngeal PCR and viral culture

**DOI:** 10.1016/j.ijid.2022.02.009

**Published:** 2022-04

**Authors:** Michalina A. Montaño, Meagan J. Bemer, Kate B. Heller, Allison Meisner, Zarna Marfatia, Elena A. Rechkina, Leah R. Padgett, Charlotte L. Ahls, Douglas Rains, Linhui Hao, Tien-Ying Hsiang, Gerard A. Cangelosi, Alexander L. Greninger, Jason L. Cantera, Allison Golden, Roger B. Peck, David S. Boyle, Michael Gale, Paul K. Drain

**Affiliations:** 1 Department of Global Health, School of Public Health, University of Washington, Seattle, WA; 2 Vaccine and Infectious Diseases Division, Fred Hutchinson Cancer Research Center, Seattle, WA; 3 Quantigen Biosciences, Fishers, IN; 4 Department of Immunology, Center for Innate Immunity and Immune Disease, University of Washington, Seattle, WA; 5 Department of Environmental and Occupational Health Sciences, School of Public Health, University of Washington, Seattle, WA; 6 Department of Laboratory Medicine and Pathology, University of Washington, Seattle, WA; 7 PATH, Seattle, WA

**Keywords:** COVID-19, SARS-CoV-2, diagnostic performance, antigen detection

## Abstract

**Objectives:**

This study assesses and compares the performance of different swab types and specimen collection sites for SARS-CoV-2 testing, to reference standard real-time reverse transcriptase-polymerase chain reaction (RT-PCR) and viral culture.

**Methods:**

Symptomatic adults with COVID-19 who visited routine COVID-19 testing sites used spun polyester and FLOQSwabs to self-collect specimens from the anterior nares and tongue. We evaluated the self-collected specimen from anterior nares and tongue swabs for the nucleocapsid (N) or spike (S) antigen of SARS-CoV-2 by RT-PCR and then compared these results with results from RT-PCR and viral cultures from nurse-collected nasopharyngeal swabs.

**Results:**

Diagnostic sensitivity was highest for RT-PCR testing conducted using specimens from the anterior nares collected on FLOQSwabs (84%; 95% CI 68-94%) and spun polyester swabs (82%; 95% CI 66-92%), compared to RT-PCR tests conducted using specimens from nasopharyngeal swabs. Relative to viral culture from nasopharyngeal swabs, diagnostic sensitivities were higher for RT-PCR and antigen testing of anterior nares swabs (91-100%) than that of tongue swabs (18-81%). Antigen testing of anterior nares swabs had higher sensitivities against viral culture (91%) than against nasopharyngeal RT-PCR (38-70%). All investigational tests had high specificity compared with nasopharyngeal RT-PCR. Spun polyester swabs are equally effective as FLOQSwabs for anterior nasal RT-PCR testing.

**Conclusions:**

We found that anterior nares specimens were more sensitive than tongue swab specimens or antigen testing for detecting SARS-CoV-2 by RT-PCR. Thus, self-collected anterior nares specimens may represent an alternative method for diagnostic SARS-CoV-2 testing in some settings.

## Highlights

• Polyester and FLOQSwabs perform in similar manner when testing for SARS-CoV-2.
• Tests using nasal swabs by RT-PCR were more sensitive than tests using tongue swabs for SARS-CoV-2.
• Self-collected nasal swabs are an accurate method for diagnosis of SARS-CoV-2.

## Introduction

The existing reference standard for the diagnosis of SARS-CoV-2 that causes COVID-19 (Gorbalenya et al., 2020; Zhu et al., 2020), is molecular detection by real-time reverse transcriptase-polymerase chain reaction (RT-PCR) assays performed in laboratories (Cheng et al., 2020; Marty, Chen, & Verrill, 2020; Tang, Schmitz, Persing, & Stratton, 2020). Although nasopharyngeal (NP) specimens collected by trained healthcare workers may yield the most sensitive test results (FDA, 2020), they may miss some SARS-CoV-2 infections (Guo et al., 2020; Wang et al., 2020). Furthermore, the deep insertion of flocked swabs into the NP space can be unpleasant for patients and must be collected by trained healthcare personnel wearing personal protective equipment (Marty et al., 2020). Supply chain disruptions and increased demand for flocked swabs during the COVID-19 pandemic have resulted in their global shortages. These challenges to specimen collection and test delivery, coupled with the increased laboratory capacity needed for scaling up COVID-19 testing, have constrained the expansion of testing capacity, particularly in resource-limited settings (Cheng et al., 2020). Identifying suitable alternative swab types, specimen types, and collection methods beyond healthcare worker-collected NP swabs for COVID-19 diagnostic tests are a priority for improving the widespread implementation of COVID-19 testing.

Detection of viral RNA by RT-PCR can quantify viral load, but may not directly indicate a live, replicating, infectious virus (Mallett et al., 2020). A current challenge is to understand when infected cases are noncontagious and yet have detectable SARS-CoV-2 by RT-PCR (Mardian, Kosasih, Karyana, Neal, & Lau, 2021; Walsh et al., 2020). A study conducted among hospitalized patients with COVID-19 reported a median duration of infectious virus shedding 8 days after symptom onset, with less than 5% probability of infectious virus shedding continuing after 15 days (van Kampen et al., 2021). Additional studies incorporating viral cultures to measure the presence of transmissible active virus report little to no viral growth beyond 8-10 days from symptom onset, suggesting that infectivity after this period is likely to be very low (Bullard et al., 2020; Singanayagam et al., 2020; Wölfel et al., 2020). RT-PCR testing can detect SARS-CoV-2 far beyond this period (Singanayagam et al., 2020), with 1 study reporting that 54% of repeat RT-PCR testing of patients already diagnosed with COVID-19 returned positive results 10-14 days after symptom onset (Mallett et al., 2020). Some patients continued to test positive over 30 days after symptom onset. However, RT-PCR cycle threshold (Ct) values are inversely related to viral load and are strongly correlated with culturability, with the probability of recovering from an infectious virus disease decreasing as Ct values increase (Singanayagam et al., 2020). Viral culture and Ct values are likely better indicators of contagiousness than qualitative RT-PCR results alone.

Identifying accurate alternatives to RT-PCR testing of NP swabs, and tests and strategies to better identify when individuals with COVID-19 are contagious, are public health priorities. The primary objective of this study was to assess the performance of different swab types (spun polyester and FLOQSwabs) and specimen collection sites (anterior nares and tongue) for SARS-CoV-2 detection by RT-PCR and nucleocapsid (N) and spike (S) antigen testing, compared with reference standard RT-PCR tests conducted using specimens from NP swabs. As a secondary objective, we compared the performance of these same investigational tests with viral cultures and their related results to NP RT-PCR Ct values and days from symptom onset.

## Methods

### Study design, setting, and population

We conducted a cross-sectional study to assess the performance of SARS-CoV-2 RT-PCR and antigen tests of different swab types and specimen collection sites. A total of 8 investigational tests were assessed RT-PCR testing of anterior nares and tongue swab samples collected on spun polyester swabs (US Cotton v3, SteriPak #60564, Lakeland, FL), RT-PCR testing of anterior nares and tongue swab specimens collected on FLOQSwabs (Copan Diagnostics, Carlsbad, CA), and N antigen and S antigen testing of anterior nares and tongue swab specimens collected on FLOQSwabs. Between July 2020, and October 2020, we recruited eligible participants who were aged 18 years or older; had any symptoms of COVID-19; and were either visiting a University of Washington (UW) Medicine COVID-19 testing site, a City of Seattle COVID-19 testing site, or had a documented recent positive COVID-19 RT-PCR result. Participants with a recent positive COVID-19 test were recruited by phone and asked to return to one of the COVID-19 testing sites to be retested and complete a study visit within 7 days of their initial test. This study was approved by the UW Institutional Review Board.

### Study visit procedures

Data on demographic characteristics, COVID-19 symptoms, and exposure were collected through electronic questionnaires administered by research staff. Owing to COVID-19 safety protocols, research staff read questions and response options aloud and recorded participant responses in electronic Research Electronic Data Capture (REDCap) data capture tools (Harris et al., 2009). Participants self-collected research swabs with guidance from research staff. Anterior nares specimens were collected, first on spun polyester and then on FLOQSwabs, by swabbing both nostrils. Tongue specimens were collected in the same order by swabbing along the length of the anterior dorsum. Clinical NP specimens were collected by clinical staff for routine RT-PCR testing, and were placed in either viral or universal transport media (VTM, UTM; Copan) and sent to the UW Clinical Virology Lab for same-day RT-PCR testing. Research swabs were stored on-site at 2°C - 8°C upon collection and transferred to a -20°C freezer at the end of each collection day. Anterior nares swabs and oral spun polyester swabs were stored without buffer, and oral FLOQSwabs were stored in Eagle's Minimum Essential Medium (EMEM, Sigma-Aldrich, St. Louis, MI). Residual UTM from clinical RT-PCR testing was stored at -80°C before transportation to a BSL-3 laboratory for SARS-CoV-2 culture.

### Laboratory procedures

Clinical NP swabs were tested using SARS-CoV-2 RT-PCR assays on Roche Cobas 6800 (Roche Molecular Diagnostics, Indianapolis, IN), the Panther System (Hologic, Inc, Marlborough, MA), or Abbott ABI7500 (Abbott Laboratories, Chicago, IL) platforms. Qualitative and quantitative clinical RT-PCR test results from NP swabs were obtained from the UW Clinical Virology Lab. Quantitative RT-PCR test results were measured as Ct values.

Anterior nares swabs were resuspended in 750 µL 1 × phosphate-buffered saline (PBS), pH 7.4, for 10 minutes at room temperature, and the supernatant was divided into 220-µL aliquots for RT-PCR and antigen testing. Tongue swab samples in EMEM were divided into 220-µL aliquots for RT-PCR and antigen testing. All aliquots were stored at -80°C until use. Anterior nares and tongue swab samples collected using FLOQSwabs were tested for SARS-CoV-2 using electrochemiluminescent (ECL) immunoassays (Debad, Glezer, Wohlstadter, & Sigal, 2004) using N and S protein assays on MesoScale Diagnostics (MSD) GOLD 96-well Small Spot Streptavidin SECTOR plates (MSD, Rockville, MD). These antigen assays used antibody pairs sourced from either Sino Biological (Wayne, PA, USA; N antibodies) or Leinco (St Louis, MI, USA; S antibodies), and recombinant N and S proteins from Acro Biosystems (Newark, DE) as reference material for creating standard curves. Plates were read and analyzed on a MESO QuickPlex SQ 120 plate reader (MSD, Rockville, MD). Samples above the lower curve fit detection range were considered positive. Samples with antigen concentration above the curve fit range were further diluted and retested. The average limit of detection (LOD) determined using the MSD Discovery Workbench software across all plates was 0.33 pg/mL and 2.33 pg/mL for N and S proteins, respectively.

The 220 µL tubes of anterior nares and tongue swab elute were tested by RT-PCR using TaqPath COVID-19 Combo Kit (Thermo Fisher) in accordance with the Emergency Use Authorization (EUA) Instructions for Use. RT-PCR was performed using the 7500 Fast Dx (Applied Biosystems, Waltham, MA), and results were analyzed using the COVID-19 Interpretive Software v1.3. RNaseP testing was performed using the TaqMan^TM^ RNaseP Assay (Thermo Fisher, Waltham, MA) on the QuantStudio DX real-time instrument with the Design and Analysis Software 2.3.3 (ABI).

Of 40 NP RT-PCR-positive samples, 32 had residual UTM available, and a SARS-CoV-2 viral culture was performed on these samples and a subset of 20 randomly selected NP RT-PCR-negative residual samples. We evaluated median tissue culture infectious dose (TCID_50_), incubation period, and plaque assays for each sample. TCID_50_ was measured using Vero E6 cells expressing human angiotensin-converting enzyme 2 and transmembrane Serine Protease 2 (VeroE6AT cells) in 96-well plates with 10,000 cells in 100 µL medium seeded into each well the day before infection. On the day of infection, 500 µL UTM was filtered with the spin column method (Corning). Seeding medium was replaced with 90 µL medium (Dulbecco's Modified Eagle Medium[DMEM] + 2% heat-inactivated fetal bovine serum [FBS] + 10 mM N-2-Hydroxyethylpiperazine-N′-2-Ethanesulfonic Acid + 1% penicillin-streptomycin) for TCID_50_ assays. A total of 10 µL of filtered UTM was added to the 90 µL medium well as the starting inoculation before 10-fold serial dilutions were performed in the second through fourth wells. Each sample was tested in triplicate and considered positive for SARS-CoV-2 viral culture if at least 1 of 3 plates showed SARS-CoV-2 growth. Cells were fixed with 10% formaldehyde and stained with 1% crystal violet 2 days after infection, digital photographs were taken, and cell death was scored. The incubation period was further assessed by measuring the time required for the virus infection to show a cytopathic effect (CPE) in the VeroE6AT cells that were seeded in 96-well plates as described previously. One day after seeding, the medium was replaced with a post-infection medium (DMEM + 2% heat-inactivated FBS + 1% L-Glu + 1% Ab/Am), and 10 µL UTM was added to each well to initiate infection. Plaque assays were performed directly from UTM samples using 10 µL inoculation of VeroE6TMPRSS2 cells and were conducted using the SARS-CoV-2/Wa1 strain (Rathe et al., 2021).

### Statistical analysis

We estimated sensitivity, specificity, positive likelihood ratio (PLR), and negative likelihood ratio (NLR) for each of the 8 investigational tests, and compared it with 2 separate reference standard tests RT-PCR and viral cultures of NP swabs. All participants contributed samples for testing by NP RT-PCR and investigational RT-PCR and antigen tests. Participants without test results or with inconclusive or invalid test results for either the investigational test or the reference standard were excluded. A stratified sample of participants, based on the results of the NP RT-PCR test, was selected for the SARS-CoV-2 culture, representing 80% of NP RT-PCR positive samples and 9.2% of NP RT-PCR negative samples. The participants whose samples were cultured were not a random sample of the study participants. For the results to be generalizable to the population and give rise to the study sample, we implemented a weighting procedure to estimate the sensitivity and specificity (Table 3). In particular, we separately reweighted NP RT-PCR positive and negative samples, an approach similar to stratified survey sampling. We used PROC SURVEYFREQ in SAS to weight the NP RT-PCR positive samples by 1.25 and the NP RT-PCR negative samples by 10.86. This analysis assumed that the participants selected for viral culture are a stratified random sample of the sample of participants who underwent NP RT-PCR testing. We estimated 95% confidence intervals for sensitivity and specificity using the binomial exact (Clopper-Pearson) method (Clopper & Pearson, 1934), and estimated 95% confidence intervals for PLR and NLR using the variance of the estimated PLR and NLR (Pepe, 2003). Median quantitative results (pg/mL) were calculated for each of the investigational antigen tests, and Wilcoxon rank-sum tests were used to compare the median quantitative antigen test results for culture-positive versus culture-negative samples.

## Results

We enrolled 261 participants between July 2020, and October 2020, of whom 217 were SARS-CoV-2-negative and 40 were SARS-CoV-2-positive according to NP RT-PCR. Four participants did not have RT-PCR results available and were not included in the analyses. Slightly more than half of the participants had been assigned female at birth, and the median age was 37 years (Table 1). Almost all participants had at least 1 COVID-19 symptom at the time of their study visit, and participants had a median of 5 days between symptom onset and study enrollment. Of 52 samples examined for SARS-CoV-2 viral culture, 11 specimens, all from NP RT-PCR-positive participants, were culture-positive, and the remaining 41 specimens were culture-negative.

**Table 1:** Characteristics of study participants.

	Positive by NP PCR (n)	Negative by NP PCR (n)	All
Total	40	217	257
			
Sex at birth			
Female	17 (42.5%)	123 (56.7%)	140 (54.5%)
Male	23 (57.5%)	94 (43.3%)	117 (45.5%)
Gender^a^			
Woman	17 (42.5%)	124 (57.1%)	141 (54.9%)
Man	23 (57.5%)	90 (41.5%)	113 (44%)
Nonbinary or genderqueer	0 (0%)	3 (1.4%)	3 (1.2%)
Race and ethnicity			
American Indian or Alaskan Native	0 (0%)	1 (0.5%)	1 (0.4%)
Native Hawaiian or Pacific Islander	1 (2.5%)	0 (0%)	1 (0.4%)
Hispanic or Latinx	3 (7.5%)	8 (3.7%)	11 (4.3%)
Asian	3 (7.5%)	16 (7.4%)	19 (7.4%)
White	22 (55%)	167 (77%)	189 (73.5%)
Black or African American	6 (15%)	6 (2.8%)	12 (4.7%)
More than one race	4 (10%)	18 (8.3%)	22 (8.6%)
Prefer not to answer	1 (2.5%)	1 (0.5%)	2 (0.8%)
Age in years (median (IQR))	34 (27)	37 (22)	37 (23)
Symptoms^b^			
No symptoms^c^	3 (7.5%)	4 (1.8%)	7 (2.7%)
One or more symptoms	37 (92.5%)	213 (98.2%)	250 (97.3%)
Days between symptom onset and enrollment (median (IQR))^d^	8 (5)	4 (5)	4 (5)
Known contact with COVID-positive people in 2 weeks before enrollment			
No	15 (37.5%)	148 (68.2%)	163 (63.4%)
Yes	20 (50.0%)	21 (9.7%)	41 (16.0%)
Unknown	5 (12.5%)	48 (22.1%)	53 (20.6%)
Hospitalization in month before enrollment			
No	39 (97.5%)	216 (99.5%)	255 (99.2%)
Yes	1 (2.5%)	1 (0.5%)	2 (0.8%)
Previous positive COVID-19 test			
No	5 (12.5%)	201 (92.6%)	206 (80.2%)
Yes	35 (87.5%)	16 (7.4%)	51 (19.8%)
Number of days between previous positive test and enrollment			
≤ 7 days	30 (75.0%)	7 (3.2%)	37 (14.4%)
> 7 days	4 (10.0%)	3 (1.4%)	7 (2.7%)
Not recruited as known positive	6 (15.0%)	207 (95.4%)	213 (82.9%)
Residual sample sent for SARS-CoV-2 viral culture^e^			
No	8 (20.0%)	197 (90.8%)	205 (79.8%)
Yes	32 (80.0%)	20 (9.2%)	52 (20.2%)

IQR: interquartile range; NP: Nasopharyngeal; RT-PCR: Reverse transcription-polymerase chain reaction; SD: standard deviation

Percentages may not add to 100 owing to rounding.

a “Woman” combines participants who responded “woman” and “trans woman.” “Man” combines participants who responded “man” and “trans man.”

b Participants could report any of the following symptoms experienced during the 2 weeks before the study visit: fever, cough, shortness of breath or difficulty breathing, chills, fatigue, shaking with chills, aches or muscle pain, runny nose, sore throat, headache, loss of taste, loss of smell, nausea, vomiting, diarrhea, dizziness or vertigo, earache, or another symptom not listed previously.

c In some cases, participants with a recent positive COVID-19 test were included despite lack of symptoms at time of visit.

d Days since symptom onset missing for 1 symptomatic participant.

e Only 32 of 40 PCR-positive samples had residual UTM available for viral culture; 20 of 217 PCR-negative samples were randomly selected for viral culture.

**Figure 1:**
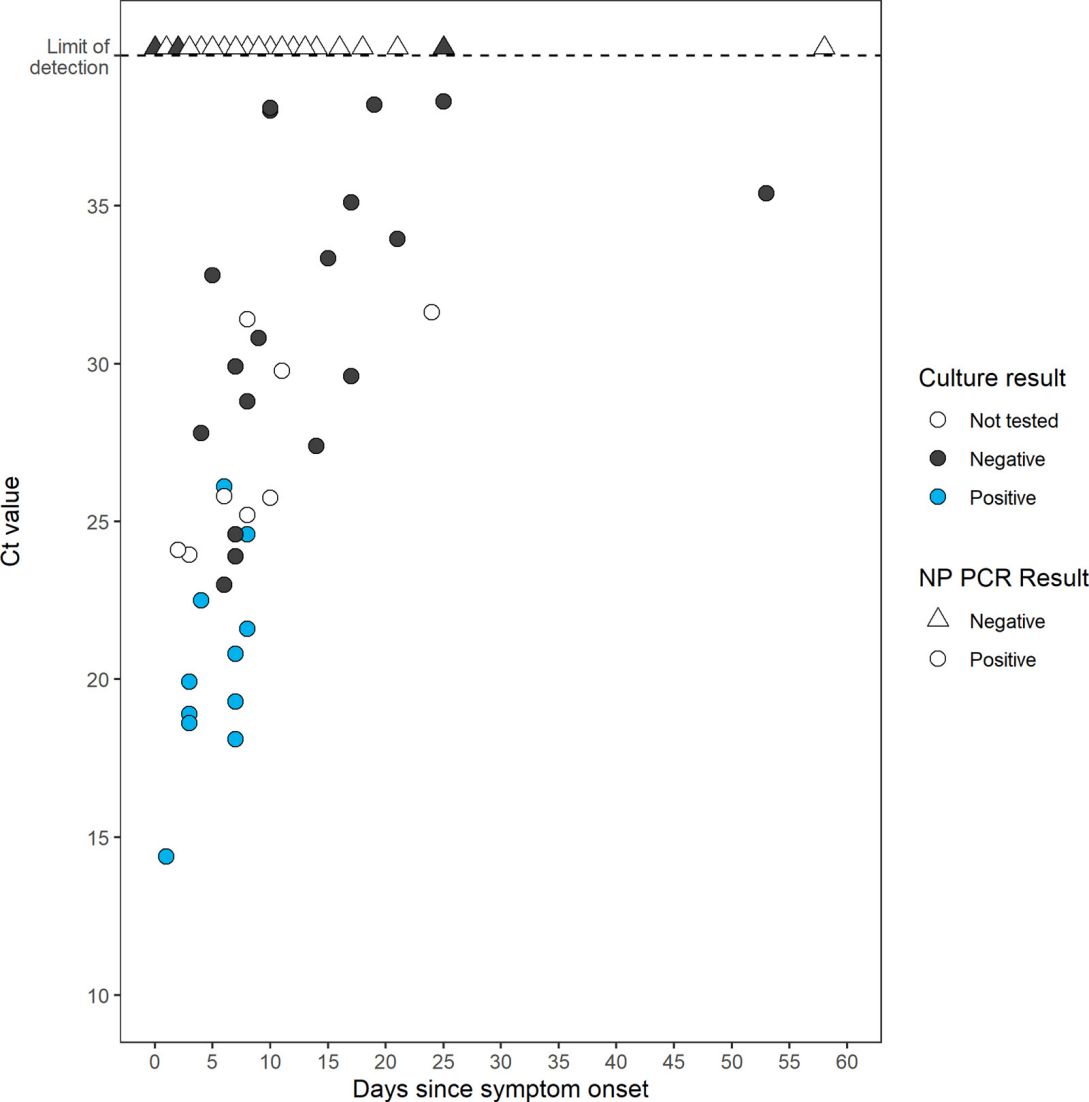
NP RT-PCR Ct value versus days since symptom onset for symptomatic participants with an NP RT-PCR result (n=249). Abbreviations: NP = nasopharyngeal; PCR = polymerase chain reaction.

**Figure 2:**
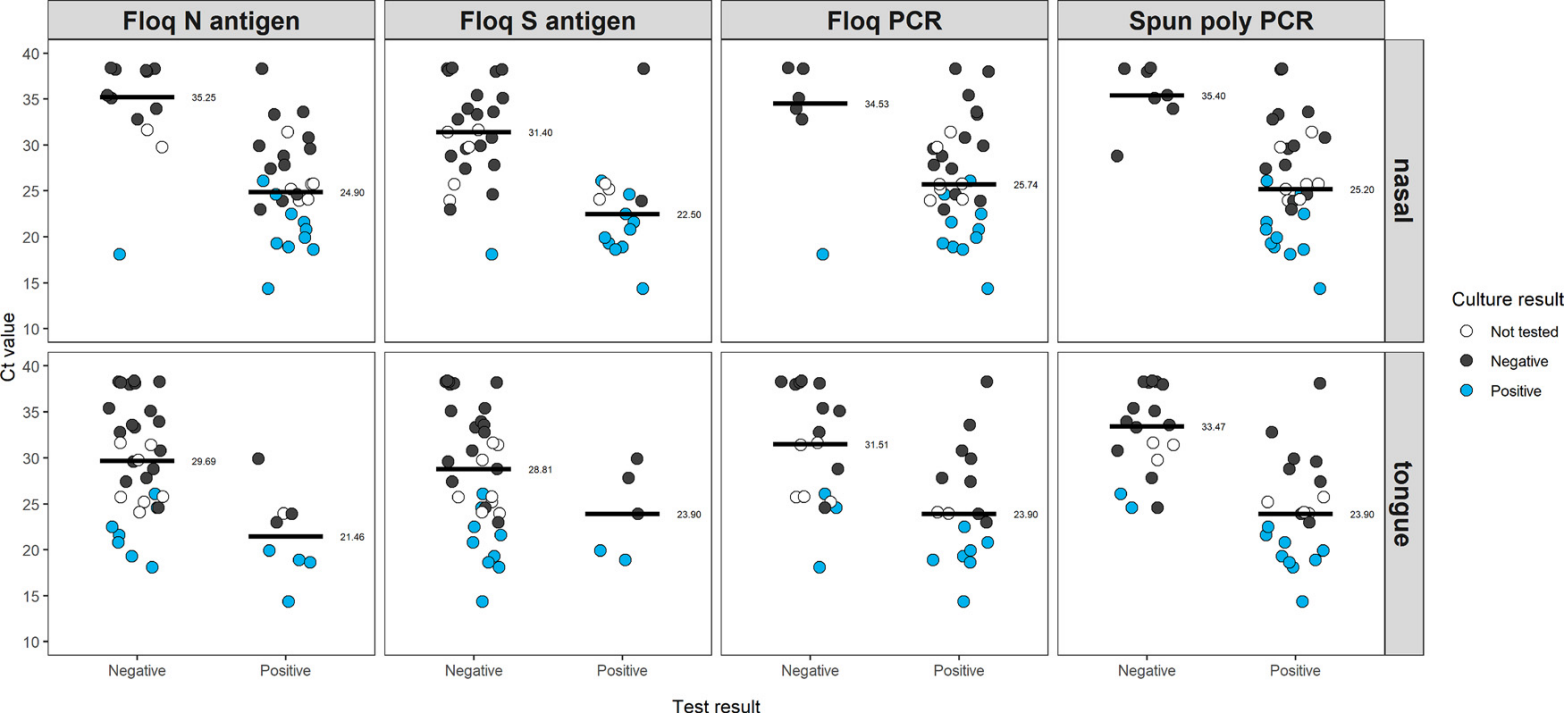
NP swab Ct value versus qualitative intervention test result. Ct values and qualitative intervention test result of all samples that were positive by NP RT-PCR. Plots are organized by sample site (anterior nares vs tongue), and intervention test (N antigen, S antigen, FLOQSwab PCR, spun polyester PCR). Black lines indicate median Ct values. To improve visibility of plots, randomized jitter of 0.15 was added in the direction of the x-axis. Abbreviations: Ct = cycle threshold; N = nucleocapsid; NP = nasopharyngeal; PCR = polymerase chain reaction; RT-PCR = reverse transcriptase-polymerase chain reaction; S = spike.

Ct values for positive NP RT-PCR test results ranged from 14.4 to 38.4 (not including those beyond LOD; Figure 1), and positive NP RT-PCR results were observed in participants with a wide range of days since symptom onset. Samples that could be cultured (culture-positive samples) came from participants on the low end of the range of days since symptom onset (1-8 days), and most had Ct values below 25. For all 8 investigational tests, the median reference standard Ct value among samples with positive investigational test results was lower than the median Ct value among samples with negative results (Figure 2).

Using NP RT-PCR results as the reference, sensitivity varied widely across the investigational tests, and was highest for anterior nares samples tested by RT-PCR, and lowest for antigen testing of tongue swabs (Table 2). Spun polyester swabs and FLOQSwabs performed equally effective. Each anterior nares swab had higher sensitivity than its tongue swab counterpart, and antigen tests were less sensitive than RT-PCR tests relative to NP RT-PCR results. Among specimens that tested positive by NP RT-PCR, there were no substantial differences in the median RnaseP value of specimens that tested positive versus negative on any of the investigational tests.

**Table 2:** Performance of the investigational testing approaches relative to the NP RT-PCR result (N=217)^a^.

	Investigational Test	TP/TP+FN	Sensitivity (95%CI)^b^	TN/TN+FP	Specificity (95%CI)^b^	Positive likelihood ratio (95% CI)^c^	Negative likelihood ratio (95% CI)^c^
**Anterior Nares Swab Samples**	RT-PCR withSpun Polyester swab	31/38	81.6 (65.7, 92.3)	212/215	98.6 (96.0, 99.7)	58.46 (18.81,181.7)	0.19 (0.10, 0.36)
	RT-PCR withFLOQSwab	31/37	83.8 (68.0, 93.8)	208/212	98.1 (95.2, 99.5)	44.41 (16.65,118.4)	0.17 (0.08, 0.34)
	N antigen	28/40	70.0 (53.5, 83.4)	214/216	99.1 (96.7, 99.9)	75.60 (18.75,304.8)	0.30 (0.19, 0.49)
	S antigen	15/40	37.5 (22.7, 54.2)	215/216	99.5 (97.4,100.0)	81.00 (11.01,596.1)	0.63 (0.49, 0.80)
**Tongue Swab Samples**	RT-PCR with SpunPolyester swab	21/39	53.8 (37.2, 69.9)	216/216	100.0 (98.3,100.0)	NC	0.46 (0.33, 0.65)
	RT-PCR withFLOQSwab	17/35	48.6 (31.4, 66.0)	216/216	100.0 (98.3,100.0)	NC	0.51 (0.37, 0.71)
	N antigen	8/40	20.0 (9.1, 35.6)	217/217	100.0 (98.3,100.0)	NC	0.80 (0.69, 0.93)
	S antigen	5/40	12.5 (4.2, 26.8)	214/217	98.6 (96.0, 99.7)	9.04 (2.25,36.34)	0.89 (0.79, 1.00)

CI: Confidence interval; FN: False Negative; FP: False Positive; NC: Not Calculable; NP: Nasopharyngeal; RT-PCR: Reverse transcription-polymerase chain reaction; TN: True Negative; TP: True Positive

^a^Row totals may sum to less than 217 owing to exclusion of inconclusive or invalid results.

b 95% CIs computed using the exact binomial (Clopper-Pearson) method.

c 95% CIs computed using the variance of the estimated PLR and NLR.

Compared with viral culture reference standard results, sensitivity was highest for anterior nares swabs, across swab types and both RT-PCR and antigen tests (Table 3). Sensitivity for tongue swabs was lower, with the lowest sensitivity observed for tongue swab antigen testing. Antigen testing of anterior nares swabs was more sensitive against reference viral culture results than against reference NP RT-PCR results. In terms of overall performance, S antigen testing of anterior nares swabs corresponded best to viral culture (10 of 11 culture-positive and 39 of 41 culture-negative results correctly identified).

**Table 3:** Performance of the investigational testing approaches relative to the NP viral culture result (N=52^a^).

	Investigational Test	TP/TP+FN	Sensitivity (95%CI)^b^	TN/TN+FP	Specificity (95%CI)^b^	Positive likelihood ratio (95% CI)^c^	Negative likelihood ratio (95% CI)^c^
**Anterior Nares Swab Samples**	RT-PCR withSpun Polyester swab	11/11	100.0 (76.5,100.0)	26/40	88.8 (74.8, 96.6)	8.93 (3.03,26.36)	0
	RT-PCR withFLOQSwab	10/11	90.9 (58.7, 99.8)	25/39	92.7 (79.7, 98.6)	12.51 (3.04,51.42)	0.10 (0.01, 0.70)
	N antigen	10/11	90.9 (58.7, 99.8)	29/41	93.8 (81.6, 98.9)	14.74 (3.52,61.80)	0.10 (0.01, 0.69)
	S antigen	10/11	90.9 (58.7, 99.8)	39/41	99.0 (89.5,100.0)	88.45 (4.04, 1936)	0.09 (0.01, 0.65)
**Tongue Swab Samples**	RT-PCR with SpunPolyester swab	9/11	81.8 (48.2, 97.7)	33/41	95.9 (84.6, 99.6)	19.90 (3.72,106.4)	0.19 (0.05, 0.76)
	RT-PCR withFLOQSwab	7/10	70.0 (34.8, 93.3)	29/37	95.6 (83.3, 99.6)	16.01 (2.73,93.93)	0.31 (0.10, 0.98)
	N antigen	4/11	36.4 (10.9, 69.2)	38/41	98.5 (88.6,100.0)	23.59 (1.36,408.8)	0.65 (0.31, 1.36)
	S antigen	2/11	18.2 (2.3, 51.8)	38/41	98.5 (88.6,100.0)	11.79 (0.24,574.5)	0.83 (0.43, 1.60)

CI: Confidence interval; FN: False Negative; FP: False Positive; NC: Not Calculable; NP: Nasopharyngeal; RT-PCR: Reverse transcription-polymerase chain reaction; TN: True Negative; TP: True Positive.

a Row totals may sum to less than 217 owing to exclusion of inconclusive or invalid results.

b 95% CIs computed using the exact binomial (Clopper-Pearson) method.

c 95% CIs computed using the variance of the estimated positive likelihood ratio (PLR) and negative likelihood ratio (NLR). NOTE: Inconclusive/invalid results excluded. Sub-sample was weighted based upon the number of NP PCR-positive and NP PCR-negative participants with viral culture results (n=20 NP PCR-negative, n=32 NP PCR-positive), respective to the total sample proportion of NP PCR results (n=217 NP PCR-negative, n=40 NP PCR-positive).

Compared with NP RT-PCR results, all 8 investigational tests had high specificity, ranging from 98-100% (Table 2). Weighted specificity relative to viral culture varied from 88.8% for anterior nares swab RT-PCR testing to 99.0% for anterior nares swab S antigen testing. The median quantitative N antigen and S antigen results from anterior nares swab testing were higher among culture-positive samples compared with culture-negative samples (N antigen: 7287.7 pg/mL vs 0.99 pg/mL, p<0.001; S antigen: 884.2 pg/mL vs. 0 pg/mL, p<0.0001). The estimated median quantitative antigen levels were 0 pg/mL among both culture-positive and culture-negative samples for both S and N antigen testing of tongue swabs (N antigen: p=0.4; S antigen: p=0.8).

## Discussion

In this comparative evaluation of different tests, swab types, and specimen types, RT-PCR tests using the anterior nares swabs were more sensitive than those using tongue swabs or antigen testing for SARS-CoV-2. Spun polyester and FLOQSwabs performed similarly across different test types and specimen types. Antigen testing, particularly S antigen testing, performed better relative to viral culture as opposed to NP RT-PCR. Among the samples that tested positive by NP RT-PCR, those that were successfully cultured had a narrower range of RT-PCR Ct values and days since symptom onset than those that were not successfully cultured.

A small number of studies have directly compared anterior nares specimens with NP specimens for the molecular detection of SARS-CoV-2. Among these, a study comparing self-collected anterior nares swabs with healthcare worker-collected NP swabs tested for SARS-CoV-2 by transcription-mediated amplification documented sensitivity of 86.3% (95%CI: 76.7-92.9%), and specificity of 99.6% (95% CI: 98.0-100.0%) (Hanson et al., 2020). A similar study, comparing the accuracy of self-collected anterior nares specimens with healthcare worker-collected NP specimens, both tested by RT-PCR, reported a sensitivity of 94% (one-sided 97.5% CI: 83.8-100.0%) for anterior nares specimens (Tu et al., 2020). The results of our comparison of self-collected anterior nares swabs versus healthcare worker-collected NP swabs are similar to the previously mentioned studies, and taken together these suggest that self-collected anterior nares swabs may be a viable alternative to NP swabs for SARS-CoV-2 testing.

Other studies have compared the accuracy of oral swabs to NP swabs by examining the performance of oropharyngeal (OP) swabs, and have reported variable results. A recent meta-analysis identified 6 studies that assessed OP versus NP swab specimens and reported that the pooled percent positive for OP swabs (84%; 95% CI: 57-100%) was similar to the pooled percent positive for NP swabs (88%; 95% CI: 73-98%), but that pooled percent agreement between OP and NP swab test results was only 68% (95% CI: 36-93%) (Lee, Herigon, Benedetti, Pollock, & Denkinger, 2021). Percent agreement between NP and OP swab results of the included studies ranged from 24% to 95%, and the use of OP swabs as an alternative to NP swabs for SARS-CoV-2 testing remains unclear. Few studies have specifically assessed the performance of tongue swabs compared with NP swabs. One of the studies reported 89.8% (97.5% CI: 78-100%) sensitivity of self-collected tongue swabs compared with healthcare worker-collected NP swabs when both were tested by RT-PCR (Tu et al., 2020). Conversely, sensitivity results from our comparison of self-collected tongue swabs to healthcare worker-collected NP swabs were closer to 50% for the 2 swab types examined. Additional study of tongue swabs as an alternate method to NP, or as a complementary noninvasive method with anterior nares swabs, will be necessary to determine the clinical relevance of this specimen type for SARS-CoV-2 testing.

Our results add to the body of evidence that spun polyester swabs perform as well as other swab types for the molecular detection of SARS-CoV-2. Two recent studies found that polyester swabs may be a suitable alternative to foam swabs, with no statistically significant differences in performance (Hart et al., 2020; Padgett et al., 2021). Another study comparing the performance of 6 different swab types found that polyester tipped swabs performed as well as other swab types for the detection of SARS-CoV-2, with no meaningful differences in viral yield (Garnett et al., 2020). As inequitable vaccine distribution continues to disproportionately impact low and middle-income countries (University, 2021; Usher, 2021), ensuring access to swabs for COVID-19 testing will continue to remain a critical component of public health response in many countries.

The results of our examination of viral cultures, days since symptom onset, and quantitative RT-PCR results are similar to previous research on this topic (Bullard et al., 2020; Singanayagam et al., 2020; Wölfel et al., 2020). We were unable to culture samples collected after 10 days from symptom onset. One study, that was able to culture samples collected later in the infection period, was conducted among patients requiring hospitalization with critical or severe COVID-19, whereas our participant population was not hospitalized at the time of sample collection (van Kampen et al., 2021). If culturability and likelihood of shedding the active virus are related to the severity of the disease, this may explain the difference in our ability to culture samples from our study population. Similar to previous findings, Ct values for participants with culturable samples tended to be lower than Ct values among participants whose samples could not be cultured in our study population (Singanayagam et al., 2020). We found that antigen testing, S antigen testing, in particular, matched up with culture results better than the other intervention tests considered. In our study population, antigen testing may have been better than RT-PCR for identifying individuals who are shedding live SARS-CoV-2 virus, supporting previous research which suggests that positive antigen tests may be good indicators of infection risk to others (Routsias, Mavrouli, Tsoplou, Dioikitopoulou, & Tsakris, 2021). If this is the case, a positive antigen test may be a better metric for determining whether an individual is contagious, and it may be useful to incorporate antigen test results for improving public health responses (Singanayagam et al., 2020).

This study had some strengths and limitations. Our study population is not a representative sample of the population accessing SARS-CoV-2 testing in Seattle because some participants were recruited based on a recent positive COVID-19 test. Owing to the demographic makeup of our population and our convenience sampling method of recruitment, these results may not be generalizable to the larger United States population—and we did not estimate positive or negative predictive values. Based on their ability to travel back to study sites for research visits, and their nonhospitalized status, our SARS-CoV-2-positive participants may have had generally milder COVID-19 disease, and this may have affected the results of RT-PCR and antigen testing, and our ability to achieve viral culture. However, the participants tested in this study may represent most unvaccinated adults being tested for SARS-CoV-2 in ambulatory sites. In addition, recruiting many of our SARS-CoV-2-positive participants from among patients with a previously documented positive RT-PCR test led to a higher median number of days since symptom onset for these participants compared with our SARS-CoV-2-negative participants. This may have impacted our ability to successfully culture samples from SARS-CoV-2-positive participants. Further, differences in the buffer used to collect clinical NP specimens, eluting dry anterior nares specimens, and collecting tongue specimens may have had some unmeasured impact on the comparison of intervention tests versus reference standard results. There may also have been uncontrollable variations in storage time at laboratories before testing. Finally, this study was conducted when different SARS-CoV-2 variants were circulating in the population, and it is unknown how these results may be transferable to new variants.

## Conclusions

In conclusion, our results add to a growing body of evidence that a range of specimen collection techniques, specimen types, and swab types return accurate SARS-CoV-2 test results compared with NP RT-PCR. This is encouraging in the face of challenges to global vaccine rollout because many countries will need to continue to increase and incorporate SARS-CoV-2 testing in their pandemic responses. The use of self-collected anterior nares swabs may be an accurate alternative method for diagnosing SARS-CoV-2 in some settings. In addition, the results of our comparison of antigen testing and viral cultures suggest that antigen tests may be useful for identifying whether individuals with COVID-19 have a replication-competent virus. Additional research on this topic will be necessary to determine the strength of the relationship between antigen test results and infectivity and quantify the ability of antigen test results to predict infectivity.

## Declarations

### Ethics approval and consent to participate

All procedures performed in this study involving human participants were in accordance with the ethical standards of the institutional and/or national research committees, and with the 1964 Helsinki declaration and its later amendments or comparable ethical standards. The study received ethical approval from the University of Washington IRB (Ref: STUDY 00009981). Written informed consent was obtained from all individual participants included in the study.

### Funding

This study was funded by the Bill and Melinda Gates Foundation, Seattle, WA.
